# Optimization of regional nodal irradiation in the era of sentinel lymph node biopsy

**DOI:** 10.20892/j.issn.2095-3941.2022.0625

**Published:** 2023-03-02

**Authors:** Zhao Bi, Xue’er Wang, Pengfei Qiu, Peng Chen, Yongsheng Wang

**Affiliations:** 1 Shandong Cancer Hospital and Institute, Shandong First Medical University and Shandong Academy of Medical Sciences, Jinan 250017, China; 2 Tianjin Medical University Cancer Institute & Hospital, National Clinical Research Center for Cancer, Key Laboratory of Cancer Prevention and Therapy, Tianjin, Tianjin’s Clinical Research Center for Cancer, Tianjin 300060, China

Historically, axillary lymph node dissection (ALND) was the standard management for axillary sentinel lymph node (SLN)-positive patients, because it enables full assessment of overall axillary lymph node (ALN) metastasis status and favorable local-regional control^1,2^. Strikingly, no differences in axillary regional recurrence or overall survival (OS) have been observed with versus without ALND among patients with early breast cancer with limited SLN involvement in several randomized, controlled trials, including the ACSOG Z0011 and AMAROS trial^3,4^. In the era of sentinel lymph node biopsy (SLNB), SLNB has replaced ALND as the standard approach for patients with 1 or 2 positive SLNs^5^. These changes in clinical practice should be considered for the optimization of regional nodal irradiation (RNI) fields.

## “Intelligent de-escalation” irradiation strategy in the era of SLNB

In the era of SLNB, the benefits of systemic therapy and radiation therapy can be combined to narrow the scope of surgery and decrease complications, thus ultimately achieving a net benefit with breast cancer treatment. Postmastectomy radiotherapy (PMRT) or RNI has advantages of avoiding ALND, but exposes patients to potentially acute or long-term toxic complications (including rare but potentially fatal second cancers and cardiac events)^6^. Decisions regarding the choice of ALND and radiotherapy must be made by a multidisciplinary team. The Memorial Sloan Kettering Cancer Center^7^ has proposed an “intelligent de-escalation” strategy in which the numbers of lymph node metastases and risk factors are used to determine whether patients should receive PMRT or RNI. The risk factors include primary factors [age < 40 years, triple negative breast cancer, and extensive lymph vascular invasion (LVI)] as well as secondary factors (hormone-receptor-positive tumor with pathology grade 3, tumor > 3 cm, high Oncotype DX index, age 40–50 years, LVI, and lymph node exocellular invasion > 2 cm). Patients with one lymph node metastasis receive PMRT or RNI if they have extensive LVI, triple negative breast cancer, age < 40 years, or 3 secondary factors. Patients with 2 lymph node metastases receive PMRT or RNI if they have any primary factors, LVI, age 40–50 years, or 2 secondary factors. However, patients with 3 lymph node metastases receive PMRT or RNI regardless of any associated risk factors. Therefore, for patients with 1 or 2 positive SLNs, the following strategies are recommended: (1) for patients receiving breast conserving surgery, ALND should be avoided, because postoperative radiation is required; (2) for patients with pN0/pNmic disease undergoing mastectomy, further axillary surgery is not acceptable; (3) if PMRT is clearly required after mastectomy for patients with 1 or 2 positive SLNs, individualized treatment should be performed according to the wishes of the patient, the occurrence of upper limb edema, radiotherapy costs and complications, the probability of non-SLN (nSLN) metastasis, and tumor burden; and (4) if the indications for PMRT are unclear after mastectomy, patients should receive ALND.

## Optimization of RNI fields in the era of SLNB

In the era of SLNB, with the widespread application of the newer approach described above, related changes in clinical practice should also be considered the optimization of radiotherapy fields. The Z0011 and AMAROS trials have shown that omission of ALND, followed by radiotherapy and adjuvant systemic therapy, is safe and produces no difference in regional recurrence in patients with early breast cancer and limited SLN involvement^3,4^. Axillary recurrence has been shown to be low, even in patients undergoing axillary de-escalation surgery, thereby suggesting that tumor biology, adjuvant systemic therapy, and radiation therapy may potentially play crucial roles^8^. Additional local control may also prolongs survival when systemic treatment is effective. In the SLNB group in the Z0011 trial, patients received high-tangential whole-breast irradiation. For patients assigned to ALND, no further axillary specific intervention and no nodal irradiation were performed^3^. In the SLNB group in the AMAROS trial, RNI included the contents of all 3 levels of the axilla as well as the medial part of the supraclavicular fossa. Adjuvant RNI after ALND was allowed when at least 4 positive nodes were found (8% of the entire population)^4^. Patients in these 2 trials received RNI in the SLNB group, whereas no RNI was administered in the ALND group. Recent data from the EBCTCG meta-analysis have indicated that the addition of RNI in women with node positive breast cancer prolongs disease-free survival by decreasing the risk of distant metastases, but does not increase OS^9^. That is, patients with positive lymph nodes may benefit from RNI. Therefore, for patients receiving ALND in the Z0011 and AMAROS trials, the overall results might have been affected if patients received adjuvant RNI after ALND. Although the Z0011 and AMAROS trials confirmed that radiotherapy can safely replace ALND for patients with 1 or 2 positive SLNs, direct evidence-based conclusions to guide the design of optimal RNI fields for these patients remained lacking. In previous studies, the probability of nSLN metastasis in patients with 1 or 2 positive SLNs was 15.9%–38.6%^2–4,10^; that is, one-third of patients with 1 or 2 positive SLNs without ALND may have additional axillary stage escalation. Therefore, the RNI fields of these patients should not be smaller than those of pN1 in patients receiving ALND.

Currently, the indications for RNI in patients receiving SLNB according to those who did receive ALND^11^. Nodal tumor burden remains an important indication for RNI in breast cancer, and accurate assessment of nodal tumor burden is important for optimizing the selection of suitable irradiation fields^10,12^. In the era of limited nodal information, decision-making regarding irradiation fields requires knowledge of lymph node involvement. In clinical practice, rather than simply referring to the Z0011 and AMAROS trial, the residual tumor burden of regional lymph nodes must be combined to determine the optimal irradiation fields. The nomograms that have been confirmed to date can predict axilla metastasis information to guide irradiation field optimization, according to actual clinical situations^10,12–15^. The most commonly used nomogram was established by the MD Anderson Cancer Center and is based on patients’ clinical and pathologic data (age, tumor burden, number of SLNs/positive SLNs, grade, and LVI)^12^. Software has been developed to calculate the probability of finding additional positive non-SLNs in patients with breast cancer with known positive SLNs. When the estimated risk of nSLN metastasis exceeds 20%–30%, RNI including the axillary and clavicle regions is strongly recommended to decrease the risk of regional recurrence. The incidence of nSLN metastasis in these patients is high, as are the risks of recurrence and metastasis. If no further ALN treatment is performed, the lymph node area (including axillary and clavicular regions) should be delineated to ensure an adequate irradiation dose of regional lymph nodes to further decrease the risk of regional recurrence.

Although the EBCTCG meta-analysis has shown that RNI improves survival for patients with positive lymph nodes, this finding was observed for the overall population (1 to 3 positive lymph nodes)^9^. According to the Memorial Sloan Kettering Cancer Center’s “intelligent de-escalation” radiotherapy strategy, some patients with positive lymph nodes may also be exempted from RNI if they have low tumor burden. These cases require consideration of nSLN metastasis risk before a comprehensive judgment is made. Therefore, patients with 1 or 2 positive SLNs without risk factors and with less than 20%–30% risk of nSLN metastasis may not require postoperative RNI; patients after breast conserving surgery would receive whole breast irradiation with or without a tumor bed boost; and patients after mastectomy would receive only chest wall radiotherapy.

## Application of internal mammary node irradiation in the era of SLNB

With continuing advances in radiotherapy technology, internal mammary node irradiation (IMNI) has improved the survival of breast cancer patients and gained increasing attention^16–18^. A meta-analysis of 3 clinical trials (EORTC 22922, MA.20, and French trial) has further confirmed that, on the basis of whole breast and chest wall irradiation, additional IMNI and supraclavicular region radiotherapy significantly prolong the 10-year OS [HR = 0.88 (95% CI 0.78–0.99)] and 10-year disease-free survival [HR = 0.86 (95% CI 0.78–0.95)]^19^. The 8-year follow-up results of the DBCG-IMN study have also shown that IMNI significantly prolongs OS [HR = 0.82 (95% CI 0.72–0.94), *P* < 0.005] and decreases mortality [HR = 0.85 (95% CI 0.73–0.98), *P* = 0.03]^20^. Survival benefits, adverse effects, and health economics should be considered during clinical application of IMNI. We believe that consideration of indications is key to the successful use of IMNI^21,22^. At present, the indications for IMNI depend primarily on the high-risk factors for internal mammary node (IMN) metastases, such as positive axillary nodes. According to extended radical mastectomy data, IMN metastasis occurs in 9.2% of patients with no positive axillary nodes, 19.6% of patients with 1–3 positive axillary nodes, and 38.3% of patients with more than 3 positive-axillary nodes^21^. The NCCN Guidelines also recommend IMNI for patients with more than 3 positive ALNs (category 1), and strongly suggest IMNI for patients with 1–3 positive ALNs (category 2A). In the era of SLNB, the indications for IMNI must also be considered in cases with unknown axillary tumor burden. Therefore, selecting populations with 1 or 2 positive SLNs in which patients could have more than 3 positive ALNs on final pathology is important. For patients with a high probability of having more than 3 positive ALNs, IMNI is recommended to decrease the risk of regional recurrence.

However, the current indications for IMNI (i.e., high risk of IMLN metastasis without histopathological confirmation of IMN) have the potential to result in over-treatment or under-treatment. Patients with high-risk factors for IMN metastasis (more than 3 positive ALNs) do not necessarily have IMN metastasis, and the possibility of IMN metastasis in low-risk patients cannot be excluded. The value of IMN metastasis information in guiding IMNI is much greater than that of high-risk factors for IMN metastases. Therefore, ensuring that only patients with IMN metastasis receive individual IMNI is important. In a subsequent prospective, multicenter CBCSG027 trial (clinical trial NCT03024463), patients with positive axillary nodes received internal mammary-SLNB (IM-SLNB) followed by first to third intercostal IMN dissection to verify the accuracy of the IM-SLNB. According to the updated CBCSG027 results, the false-negative rate of IM-SLNB was 3.28%, and the internal mammary-SLN was the only positive IMN in 52.5% of patients^22^. Positive IMNs were found in 61 (41.2%) of 148 patients. Moreover, 72.0% of patients had axillary pN1, and 42.4% of those with axillary pN2-3 could safely avoid IMNI after a negative IM-SLNB. A nomogram for the prediction of IMN metastasis (AUC of 0.860) has also been produced to guide the use of IMNI. The relevant research results will be reported in the future.

In conclusion, in the era of SLNB, irradiation field decision-making must consider the residual tumor burden of regional lymph nodes to achieve a net benefit of breast cancer treatment.

## References

[1] Ozcan LC, Giuliano AE (2017). Is axillary lymph node dissection necessary after a positive sentinel lymph node biopsy?. Adv Surg.

[2] Krag DN, Anderson SJ, Julian TB, Brown AM, Harlow SP, Costantino JP (2010). Sentinel-lymph-node resection compared with conventional axillary-lymph-node dissection in clinically node-negative patients with breast cancer: overall survival findings from the NSABP B-32 randomized phase 3 trial. Lancet Oncol.

[3] Giuliano AE, Ballman K, McCall L, Beitsch P, Whitworth PW, Blumencranz P (2016). Locoregional recurrence after sentinel lymph node dissection with or without axillary dissection in patients with sentinel lymph node metastases: long-term follow-up from the American College of Surgeon Oncology Group (Alliance) ACOSOG Z0011 Randomized Trial. Ann Surg.

[4] Donker M, van Tienhoven G, Straver ME, Meijnen P, van de Velde CJ, Mansel RE (2014). Radiotherapy or surgery of the axilla after a positive sentinel node in breast cancer (EORTC 10981-22023 AMAROS): a randomized, multicenter, open-label, phase 3 non-inferiority trial. Lancet Oncol.

[5] Li JJ, Cheng JY, Liu GY, Hou Y, Di G, Yang B (2022). Feasibility of sentinel lymph node biopsy omission after integration of ^18^F-FDG dedicated lymph node PET in early breast cancer: a prospective phase II trial. Cancer Biol Med.

[6] Jagsi R, Chadha M, Moni J, Ballman K, Laurie F, Buchholz TA (2014). Radiation field design in the ACOSOG Z0011 (Alliance) trial. J Clin Oncol.

[7] Morrow M (2022).

[8] Bi Z, Chen JJ, Liu PC, Chen P, Wang WL, Liu YB (2021). Candidates of genomic tests in HR+/HER2-breast cancer patients with 1-2 positive sentinel lymph node without axillary lymph node dissection: analysis from multicentric cohorts. Front Oncol.

[9] McGale P, Taylor C, Correa C, Cutter D, Duane F, EBCTCG (Early Breast Cancer Trialists’ Collaborative Group) (2014). Effect of radiotherapy after mastectomy and axillary surgery on 10-year recurrence and 20-year breast cancer mortality: meta-analysis of individual patient data for 8135 women in 22 randomised trials. Lancet.

[10] Yang ZB, Lan XW, Huang Z, Yang Y, Tang Y, Jing H (2020). Development and external validation of a nomogram to predict four or more positive nodes in breast cancer patients with one to three positive sentinel lymph nodes. Breast.

[11] Wang YJ, Chen JY (2018). Radiotherapeutic strategies for breast cancer patients in accordance with ACOSOG Z0011 study criteria. Chin J Pract Surg.

[12] Katz A, Smith BL, Golshan M, Niemierko A, Kobayashi W, Raad RA (2008). Nomogram for the prediction of having four or more involved nodes for sentinel lymph node-positive breast cancer. J Clin Oncol.

[13] Xie X, Tan W, Chen B, Huang X, Peng C, Yan S (2018). Preoperative prediction nomogram based on primary tumor miRNAs signature and clinical-related features for axillary lymph node metastasis in early-stage invasive breast cancer. Int J Cancer.

[14] Sa-Nguanraksa D, O-Charoenrat E, Kulprom A, Samarnthai N, Lohsiriwat V, Nimpoonsri K (2019). Nomogram to predict non-sentinel lymph node status using total tumor load determined by one-step nucleic acid amplification: first report from Thailand. Breast Cancer.

[15] Unal B, Gur AS, Beriwal S, Tang G, Johnson R, Ahrendt G (2009). Predicting likelihood of having four or more positive nodes in patient with sentinel lymph node-positive breast cancer: a nomogram validation study. Int J Radiat Oncol Biol Phys.

[16] Whelan TJ, Olivotto IA, Parulekar WR, Ackerman I, Chua BH, Nabid A (2015). Regional nodal irradiation in early-stage breast cancer. N Engl J Med.

[17] Poortmans PM, Collette S, Kirkove C, Van Limbergen E, Budach V, Struikmans H (2015). Internal mammary and medial supraclavicular irradiation in breast cancer. N Engl J Med.

[18] Poortmans PM, Weltens C, Fortpied C, Kirkove C, Peignaux-Casasnovas K, Budach V (2020). Internal mammary and medial supraclavicular lymph node chain irradiation in stage I-III breast cancer (EORTC 22922/10925): 15-year results of a randomised, phase 3 trial. Lancet Oncol.

[19] Budach W, Bölke E, Kammers K, Gerber PA, Nestle-Krämling C, Matuschek C (2015). Adjuvant radiation therapy of regional lymph nodes in breast cancer - a meta-analysis of randomized trials-an update. Radiat Oncol.

[20] Thorsen LB, Offersen BV, Danø H, Berg M, Jensen I, Pedersen AN (2016). DBCG-IMN: a population-based cohort study on the effect of internal mammary node irradiation in early node-positive breast cancer. J Clin Oncol.

[21] Qiu PF, Wang XE, Wang YS (2021). Indications for individual internal mammary node irradiation. Lancet Oncol.

[22] Wang Y, Lv Q, Zhu S, Zhao W, Yang G, Huang Y (2020). Cancer Res.

